# Multibacillary leprosy patients with high and persistent serum antibodies to leprosy IDRI diagnostic-1/LID-1: higher susceptibility to develop type 2 reactions

**DOI:** 10.1590/0074-02760150198

**Published:** 2015-11

**Authors:** Danielle de Freitas Mizoguti, Emerith Mayra Hungria, Aline Araújo Freitas, Regiane Morillas Oliveira, Ludimila Paula Vaz Cardoso, Mauricio Barcelos Costa, Ana Lúcia Maroclo Sousa, Malcolm S Duthie, Mariane Martins Araújo Stefani

**Affiliations:** 1 Universidade Federal de Goiás, Instituto de Patologia Tropical e Saúde Pública, Goiânia, GO, Brasil; 2 Infectious Disease Research Institute, Seattle, WA, USA

**Keywords:** antibody, diagnosis, LID-1, leprosy reactions, serology, PGL-I

## Abstract

Leprosy inflammatory episodes [type 1 (T1R) and type 2 (T2R) reactions] represent the major cause of irreversible nerve damage. Leprosy serology is known to be influenced by the patient's bacterial index (BI) with higher positivity in multibacillary patients (MB) and specific multidrug therapy (MDT) reduces antibody production. This study evaluated by ELISA antibody responses to leprosy Infectious Disease Research Institute diagnostic-1 (LID-1) fusion protein and phenolic glycolipid I (PGL-I) in 100 paired serum samples of 50 MB patients collected in the presence/absence of reactions and in nonreactional patients before/after MDT. Patients who presented T2R had a median BI of 3+, while MB patients with T1R and nonreactional patients had median BI of 2.5+ (p > 0.05). Anti-LID-1 and anti-PGL-I antibodies declined in patients diagnosed during T1R (p < 0.05). Anti-LID-1 levels waned in MB with T2R at diagnosis and nonreactional MB patients (p < 0.05). Higher anti-LID-1 levels were seen in patients with T2R at diagnosis (vs. patients with T1R at diagnosis, p = 0.008; vs. nonreactional patients, p = 0.020) and in patients with T2R during MDT (vs. nonreactional MB, p = 0.020). In MB patients, high and persistent anti-LID-1 antibody levels might be a useful tool for clinicians to predict which patients are more susceptible to develop leprosy T2R.

Leprosy is a chronic, granulomatous disease that results from infection with*Mycobacterium leprae*. Although the disease affects the skin and peripheral nerves, it can present with a wide array of pathologies and clinical manifestations depending upon the patient's immune response (Scollard et al. 2006). At the tuberculoid (TT) pole, patients develop strong cell-mediated immunity (CMI) to *M. leprae* characterised by a T-helper (Th)1 type response with interferon-gamma secretion that results in low bacillary load, few skin lesions and low or absent antibody production. The lepromatous (LL) pole is characterised by low or absent *M. leprae-*specific CMI, but vigorous antibody production, high bacillary loads and multiple disseminated skin lesions. The intermediary borderline forms (borderline tuberculoid, borderline borderline and borderline lepromatous) may show immunological changes towards either pole of the spectrum (Ridley & Jopling 1966). For operational purposes, a simplified classification system based on counting the number of skin lesions was proposed by the World Health Organization (WHO): patients with up to five skin lesions are considered paucibacillary (PB) and patients with more than five skin lesions are considered multibacillary (MB). PB and MB patients are prescribed with different multidrug therapy (MDT) regimens consisting of daily treatment for six or 12 doses, respectively (WHO 1991).

During the course of the disease and even during treatment, a significant proportion of patients develop acute inflammatory complications known as type 1 (T1R) and type 2 (T2R) reactions. Because they can cause irreversible nerve damage, leprosy reactions represent the major cause of permanent physical disabilities and deformities (Richardus et al. 2004, Illarramendi et al. 2012). T1R is associated with alterations in Th1 type CMI while T2R is associated with immune complex deposition and transient CMI activation (Kahawita et al. 2008). Identifying markers or correlates of leprosy reactions could allow the tailored management of patients at higher risk of developing reactions and help to distinguish them earlier. No laboratory assays are currently used to identify or predict the risk of developing reactional episodes.

The detection of IgM antibodies against phenolic glycolipid I (PGL-I) represents the most evaluated serologic assay for leprosy, with levels correlating with bacillary loads such that levels rise across the TT to LL spectrum (Moura et al. 2008). Although conflicting observations have been made, several studies have indicated high anti-PGL-I levels as risk factors for the development of both types of leprosy reactions (Roche et al. 1993, 1997, Stefani et al. 1998, Brito et al. 2008). Following the publication of the *M. leprae* genome more than a decade ago, more than 200 protein antigens have been evaluated in immunological assays (Cole et al. 2001, Spencer et al. 2005, Aráoz et al. 2006, Stefani 2008, Geluk et al. 2009, 2010). The leprosy Infectious Disease Research Institute diagnostic-1 (LID-1) fusion protein, which combines the ML0405 and ML2331 gene products into a single molecule, is well recognised by IgG antibodies in the serum of MB patients from numerous leprosy-endemic regions (Reece et al. 2006, Duthie et al. 2007, 2010,Sampaio et al. 2011, Hungria et al. 2012). We therefore evaluated the potential of using serum antibody responses against new protein antigens of *M. leprae *for the diagnosis or prognosis of leprosy reactions.

Previous studies have shown that IgM and IgG leprosy serology is influenced by the patient's bacterial index (BI) with higher positivity towards MB disease (Bührer-Sekula et al. 2000, Duthie et al. 2010, Hungria et al. 2012). Moreover, MDT has been shown to reduce *M. leprae*-specific serum antibody responses. To consider the impact of both MDT and the immunosuppressive treatments of leprosy reactions, which respectively has been shown to reduce antibody levels, MB patients were stratified according to the type of leprosy reaction (T1R and T2R) and the time of occurrence of reactions (at diagnosis or during MDT). Our data indicate that responses to LID-1 are highest in patients presenting with T2R and that the persistence of anti-LID-1 antibodies during treatment indicates patients more susceptible to develop T2R.

## SUBJECTS, MATERIALS AND METHODS

*Patients* - This study was approved by the Research Ethical Committee of the Clinics Hospital, Federal University of Goiás (UFG), Goiânia, state of Goiás, and by the Brazilian Research Ethics Commission, with all participants signing an informed consent before enrolment. A retrospective analytical study was conducted with 50 patients that were recruited at the time of leprosy diagnosis and monitored during MDT for the development of leprosy reactions at the main regional outpatient clinic (Reference Center for Diagnosis and Treatment, Goiânia).

Newly diagnosed, untreated MB leprosy patients (determined by WHO operational criteria; both genders, no age restrictions) were recruited as presenting with or without reaction, then fully characterised according to Ridley and Jopling criteria considering clinical, bacilloscopic and histopathology analyses (Table). Patients were then provided standard WHO-MDT and monitored for the development of reactions. For reaction-free patients, blood was collected at the time of initial diagnosis and at the end of MDT. For patients that presented reactional episode at diagnosis, blood was collected at diagnosis and at the end of reactional episode; for reactional patients that were reaction-free at diagnosis, blood sample was collected during the occurrence of reactional episode on follow up. Therefore, 100 serum samples were prepared and stored at -20ºC until analyses.

**TABLE:** Characteristics of the study participants

Study groups	n	Gender (M/F)	Age years [median (range)]	Ridley and Jopling classification	BI [median (range)]
No reaction	12	6/6	51 (20-65)	2 BT/1 BB/6 BL/3 LL	2.5 (0-6)
T1R	26	21/5	50 (19-79)	5 BT/4 BB/17 BL/0 LL	2.5 (1-5)
T2R	12	11/1	35 (17-58)	0 BB/2 BL/10 LL	3 (1-5)
Total	50	38/12	47 (17-79)	7 BT/5 BB/25 BL/13 LL	3 (0-6)

BB: borderline borderline; BI: bacillary index; BL: borderline lepromatous; BT: borderline tuberculoid; F: female; LL: lepromatous; M: male; T1R: type 1 reaction; T2R: type 2 reaction.

*Antigen-specific antibody detection* - Serum IgM antibodies to*M. leprae *PGL-I were detected by ELISA. Briefly, polysorp 96-well plates (Nunc Maxisorp) were coated with 0.01 μg/mL natural trisaccharide-phenyl conjugated to bovine serum albumin (NT-P-BSA), the trisaccharide synthetic analog of PGL-I kindly provided by Dr Fujiwara, Nara University, Japan, and blocked with phosphate-buffered saline (PBS)-1% BSA. Serum samples diluted 1/300 in 1% BSA were added to duplicate wells of either NT-P-BSA or BSA-coated plates. After incubation for 1 h at 37ºC and washing with PBS-Tween, horseradish peroxidase-conjugated to anti-human IgM (Jackson ImmunoResearch, USA) was then added. After incubation for 1 h at 37ºC and further washes, 3,3′,5,5′-tetramethylbenzidine peroxidase colour substrate (Sigma) was added for each well. The colour reactions of the entire plate were stopped with 2.5 N-H_2_SO_4_. The optical density (OD) was read at 450 nm using a Multiskan Ex microplate reader (Thermo Scientific, USA). The results were expressed as mean absorbance of the duplicates. The final OD value of each serum sample was calculated by subtracting the OD value of wells coated only with BSA from the OD value of the test wells coated with NT-P-BSA. The cut-off was defined as OD > 0.250 in accordance with Bührer et al. (1998).

Serum IgG antibodies to the di-fusion protein LID-1 were detected by ELISA. Polysorp 96-well plates (Nunc Maxisorp) were coated with 2 μg/mL of LID-1 at 4ºC overnight and blocked with PBST with 1% BSA for 1 h at room temperature (RT). Serum samples diluted 1/200 in 0.1% PBS-BSA were added in duplicates and incubated for 2 h at RT. Plates were washed and incubated with 100 μL of peroxidase-conjugated with anti-human IgG (Sigma) diluted to 1/5,000 in PBST, 0.1% BSA. After washings, reactions were developed with peroxidase colour substrate (KPL, USA) and quenched by the addition of 1N-H_2_SO_4_. The corrected OD of each well at 450 nm was read using a Multiskan Ex microplate reader. Based on previous data, the threshold for positive responses was calculated as 2x standard deviation of the OD of sera from healthy endemic controls, such that samples with OD > 0.3 were considered positive (Duthie et al. 2007).

*Statistical analyses* - GraphPad Prism v.5 was used for the calculation of the median and mean values of OD and for graphics. Statistical significance was assessed by Kruskal-Wallis one way analysis of variance for comparison of multiple groups and Mann-Whitney *U* for comparison between two groups. Results were considered statistically significant when p values < 0.05 were obtained.

*Ethics* - This study was approved by the Ethical Committee of the Clinics Hospital/UFG (protocol 456.226). All participants were informed about the study aims and the procedures involved, then included only after signing the Informed Consent Form in accordance with Resolution 196/1996 of the National Health Council.

## RESULTS

**Fig. 1:**
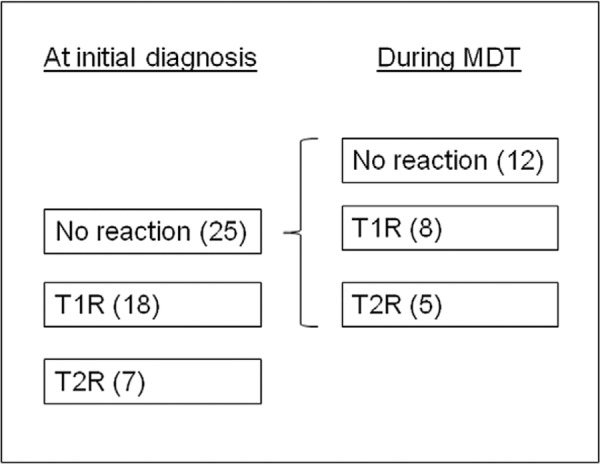
recruitment and stratified groups based on presentation and evolution of disease. MDT: multidrug therapy; T1R: type 1 reaction; T2R: type 2 reaction.

*Patient demographics at time of initial diagnosis* - The study group was composed by 50 MB patients with ages ranging from 17-79 years (median = 47 years) of whom the majority was male (Table). Stratification was then conducted based on the type of reactional episode at the time of initial diagnosis (none, T1R or T2R) (Fig. 1). Within this study group a half of MB patients either presented with T1R or T2R at the time of initial diagnosis (25 of 50) (Fig. 1). The only demographic difference in subgroups at the time of presentation was that patients who presented with T2R were significantly younger than the nonreactional MB patients (p = 0.026)*.* In this study group, MB patients who presented with T2R had a median BI of 3+, while MB patients with T1R and nonreactional patients had median BI of 2.5+ (p > 0.05). Although the patients presenting with T2R were predominantly LL, the BIs of MB reactional patients and reaction-free MB patients were similar (p > 0.05).

**Fig. 2:**
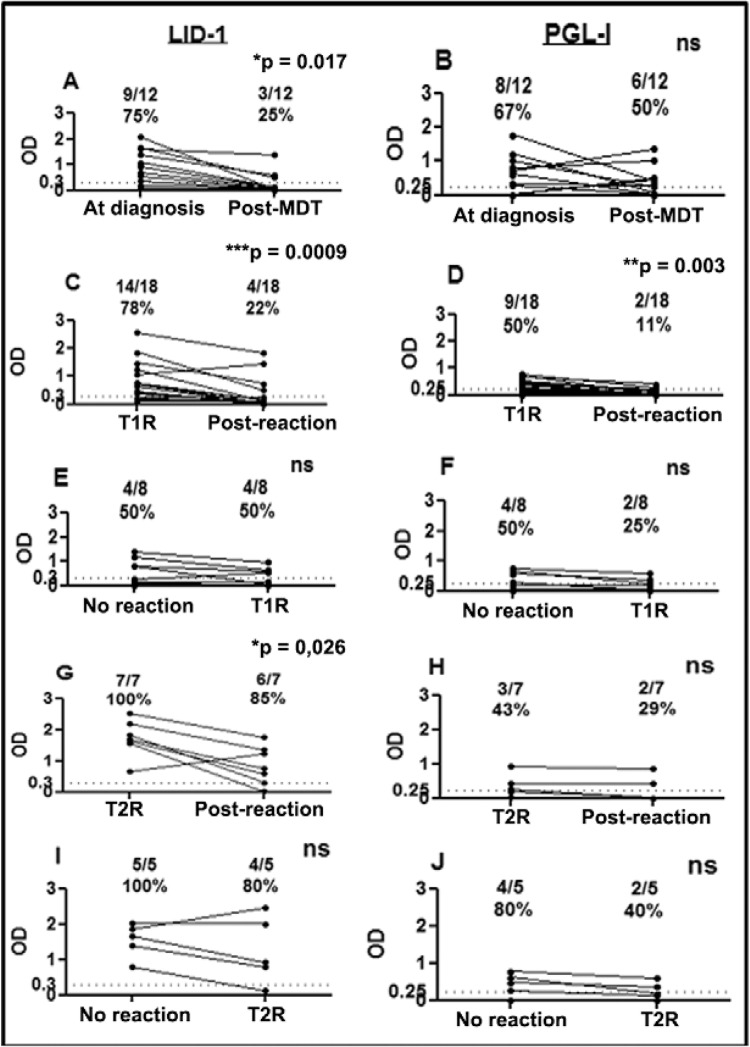
serological reactivity to leprosy Infectious Disease Research Institute diagnostic-1 (LID-1) and to phenolic glycolipid I (PGL-I) in paired serum samples from multibacillary (MB) patients who developed type 1 (T1R) and type 2 (T2R) reactions at diagnosis or during multidrug therapy (MDT) and among nonreactional MB (nonreactional MB patients: n = 12). A; seroreactivity to LID-1; B: seroreactivity to PGL-I (MB patients who developed T1R at diagnosis: n = 18); C: seroreactivity to LID-1; D: seroreactivity to PGL-I MB patients who developed T1R during MDT (n = 5); E: seroreactivity to LID-1; F: seroreactivity to PGL-I (MB patients who developed T2R at diagnosis: n = 7); G: seroreactivity to LID-1; H: seroreactivity to PGL-I (MB patients who developed T2R during MDT: n = 5); I; seroreactivity to LID-1; J: seroreactivity to PGL-I. For nonreactional patients paired samples were collected at diagnosis and after MDT. For reactional patients, each point represents the optical density (OD) in each sample taken from the same patient in the presence and in the absence of the reaction. The dashed line represents the cut-off: OD > 0.3 to anti-LID-1 and OD > 0.25 to anti-PGL-I serology. Asterisks mean p < 0.05. ns: not statistically significant.

**Fig. 3:**
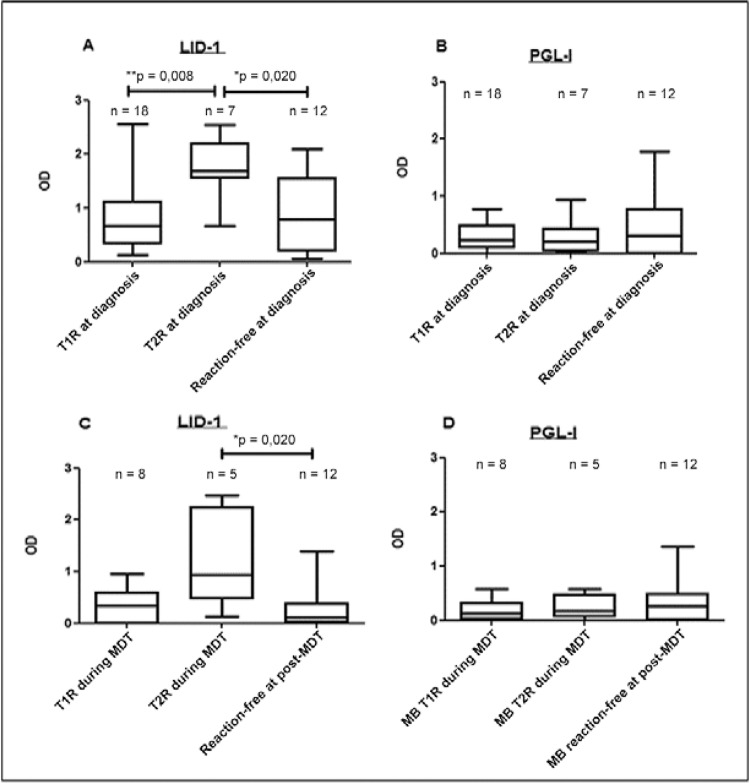
antibody levels against: leprosy Infectious Disease Research Institute diagnostic-1 (LID-1) (A), phenolic glycolipid I (PGL-I) (B) in multibacillary (MB) patients who presented at the time of initial diagnosis with either type 1 reaction (T1R) (n = 18), type 2 reaction (T2R) (n = 7) or no reaction (n = 12). Antibody responses to LID-1 (C) and to PGL-I (D) in MB patients who presented during multidrug therapy (MDT) with either T1R (n = 8), T2R (n = 5) or no reaction (n = 12). The boxes represent the 25th and 75th percentiles for each group, while lines in the box mark the median optical density (OD). Asterisks mean p < 0.05.

*Antibody responses at time of initial diagnosis* - As expected, at the time of diagnosis, the vast majority of MB patients presented with positive anti-LID-1 and anti-PGL-I responses (81% and 54 %, respectively). The rate of seropositivity among nonreactional MB patients was 75% (9/12) for anti-LID-1 and 67% (8/12) for anti-PGL-I antibodies (Fig. 2A,B). Similarly, among MB patients 78% (14 of 18) exhibiting T1R at the time of diagnosis recognised LID-1 antigen and 50% (9 of 18) was anti-PGL-I positive (Fig. 2C, D). The highest rate of anti-LID-1 seropositivity was observed in patients presenting with T2R, with all seven (100%) seropositive for LID-1 (Fig. 2G) while anti-PGL-I responses in patients presenting with T2R was 43% (3/7). In addition to the rate of anti-LID-1 positivity being greater, patients presenting with T2R also had higher levels of anti-LID-1 antibodies when compared to both nonreactional and T1R patients (Fig. 3A) (p = 0.020 and 0.008, respectively). Anti-PGL-I responses were similar in these same groups of MB patients (Fig. 3B). Thus, high levels of anti-LID-1 at the time of diagnosis were indicative of a T2R.

Compared to serological reactivity observed during the T1R episode, antibody levels to both LID-1 and PGL-I dropped after resolution (p = 0.0009 and 0.003, respectively) (Fig. 2C, D). In contrast, while LID-1 seropositivity decreased significantly after resolution of T2R (p = 0.026) (Fig. 2G), 29% of these T2R patients remained anti-PGL-I positive after resolution [vs. 43% at diagnosis (Fig. 2H), p*> *0.05]. Together, these data indicate that antibody responses generally decline following treatment of the reactional episode.

*Antibody responses during MDT* - The rate of seropositivity against LID-1 among patients who did not have reactions was 75% (9 of 12). Although 25% (3 of 12) were still seropositive after completing MDT, the magnitude of anti-LID-1 response of the seropositive patients was lower relative to before the commencement of MDT (Fig. 2A) (p = 0.017). In these same patients, compared to anti-LID-1 responses, the seropositivity to PGL-I was marginally lower at 67% (8 of 12) at diagnosis, but higher at 50% (6 of 12) after MDT (p > 0.05). The anti-PGL-I response actually rose in two of the eight patients who were seropositive at diagnosis and two additional seropositive individuals emerged from the group that was seronegative at the time of diagnosis (Fig. 2B). Thus, in MB patients that did not have reactions, compared to anti-PGL-I responses, the anti-LID-1 response demonstrated a more consistent decline during MDT.

Of the 25 MB patients who were reaction-free at leprosy diagnosis, 26% (13 of 25) subsequently developed them during MDT (8 developed T1R and 5 developed T2R). For MB patients who developed T1R during MDT (n = 8), 50% patients were seropositive to LID-1 at time of diagnosis and at time of reaction (Fig. 2E) (p *> *0.05). Anti-PGL-I responses of these patients before and during reactions were similar (Fig. 2F) (p > 0.05).

All MB patients who developed T2R during the MDT were seropositive to LID-1 at the time of diagnosis and no significant reduction in seropositivity was observed at the time of T2R (Fig. 2I) (p *>*0.05). Similarly, 80% of this subset were seropositive against PGL-I at the time of diagnosis and although this was reduced to 40% at the time of T2R, this change was not significant (Fig. 2J) (p*> *0.05).

Among MB patients who developed leprosy reaction during MDT, patients presenting with T2R showed higher levels of anti-LID-1 antibodies when compared to nonreactional MB patients (p *= *0.020), but not when compared to patients who developed T1R (Fig. 3C). Anti-PGL-I responses were similar in these same groups of MB patients (Fig. 3D).

*Comparative analyses of anti-LID-1 and anti-PGL-I responses* - In comparative analyses of anti-LID-1 and anti-PGL-I responses in patients that developed T2R at diagnosis or during MDT, the only statistically significant difference was a higher number of positive anti-LID-1 responses in patients that developed T2R at diagnosis (p = 0.03 and p = 0.13 respectively). All seven patients that were diagnosed during a T2R episode were anti-LID-1 positive and three out of them were anti-PGL-I positive. Compared to anti-PGL-I positivity, a higher number of anti-LID-1 positives was seen in patients with T1R at diagnosis and with T1R during MDT, however these differences were not statistically significant (p = 0.083 and p = 0.533 respectively). For the 18 patients diagnosed during a T1R episode, 14 were anti-LID-1 positive and nine were anti-PGL-I positive. Eight out of nine PGL-I positives were LID-1 positives.

## DISCUSSION

Leprosy reactions can be a major complication for leprosy patients and are a major cause of permanent disability and incapacities. Reactions are acute immune inflammatory episodes that are characterised by dysregulated and exacerbated immune responses to *M. leprae* and up to now there is no laboratory marker or predictor of these events. The key findings of this study were that anti-LID-1 IgG levels were highest in MB patients first diagnosed during a T2R and that anti-LID-1 levels persisted in patients that developed reactions during MDT. These results indicate that high and persistent levels of anti-LID-1 may be associated with the occurrence of T2R, regardless of whether the episode occurs at diagnosis or during MDT.

Recent studies have addressed potential applications of anti-LID-1 serology. A rapid test using PGL-I mimetic, ND-O and LID-1 antigens as a single fusion complex (ND-O-LID) impregnated on a nitrocellulose membrane has been developed as a simple and fast system that can be used with a minimal amount of training to provide an objective diagnosis of MB leprosy (Cardoso et al. 2013). The decline in IgG responses to LID-1 after MDT was shown in some studies indicating its potential use to monitor MDT (Duthie et al. 2011, Rada et al. 2012, Freitas et al. 2015). Studies from different areas in Brazil (states of Minas Gerais and Paraíba) have confirmed anti-LID-1 serology as a tool for the detection of MB leprosy and for the identification of individuals with subclinical infection (de Souza et al. 2014, Fabri et al. 2015). A study using ND-O-LID and LID-1 to investigate antibody responses of leprosy patients from Colombia and the Philippines in a rapid ELISA assay system indicated correlation with bacteriological index, suggesting its use to replace skin slit smears (Duthie et al. 2014). In difficult-to-reach mountain areas in Southwest China, elevations in anti-LID-1 and anti-PGL-I responses on sequential samples of contacts of leprosy patients was used for the early diagnosis of MB leprosy (Qiong-Hua et al. 2013). The current study adds another potential application of anti-LID-1 serology as a tool to discriminate MB patients who can be more susceptible for the development of T2R reactions.

Confirming previous reports, our examination of anti-PGL-I responses among MB patients who developed T1R or T2R did not reveal detectable differences compared to nonreactional MB patients (Roche et al. 1993). The decline of anti-PGL-I antibodies in MB patients with T1R at diagnosis was significant compared to MB patients who developed leprosy reactions during MDT. Given the already described effect of MDT reducing antibody titres, we cannot rule out the influence of treatment in the fluctuation of antibody levels in leprosy reactions that occurred during MDT. A previous case-control study has shown that patients who had positive anti-PGL-I serology that persisted after treatment had a higher risk to develop leprosy reaction when compared to those who became seronegative (Brito et al. 2008). In our study, most reaction-free MB patients had high anti-PGL-I levels even after MDT similarly suggesting that these patients may be more susceptible to develop reactions and indicating the need for continued monitoring.

The serological response to recombinant proteins of* M. leprae *during leprosy reaction has not been described; however, previous serological analyses have indicated antigen-specific IgG levels reflect the bacterial load of the patient, similarly to what has been shown in IgM anti-PGL-I serology (Duthie et al. 2007, 2008,2010). In this sense, it is noteworthy that, despite all MB patients in the study having similar bacterial indices, patients who developed T2R showed higher anti-LID-1 antibodies levels.

Compared to PGL-I, higher positivity to anti-LID-1 was seen in patients that developed T2R at diagnosis indicating that LID-1 serology seemed more effective to discriminate these patients. Although a higher number of anti-LID-1 positivity was seen in patients with T1R (at diagnosis or during MDT) these differences were not statistically significant. Further studies on the correlation of PGL-I and LID-1 responses in leprosy reactional patients may validate these findings. It is possible that higher anti-LID-1 responses compared to PGL-I may be, at least partially, due to the fact that LID-1 is a fusion protein and responses to it represent the sum of serologic responses to two highly immunogenic proteins ML0405 and ML2331.

An additional clinical finding in our study was that MB patients who developed T2R were younger than nonreactional MB patients (median ages 35 for T2R and 51 for nonreactional MB patients). A previous study that evaluated risk factors for the development of T2R showed a decreased risk in patients older than 40 years (Manandhar et al. 1999). For T1R, older age (≥ 15 years) at leprosy diagnosis was shown to be a strong independent risk factor among Vietnamese patients (Ranque et al. 2007). Compared to nonreactional MB patients, the younger age among patients that develop T2R suggests that a genetic component may be also involved in this susceptibility at younger age. Thus, our data suggest that younger MB patients with high anti-LID-1 antibody levels should be closely monitored for the development of T2R.

The significant drop in the antibody levels observed during treatment has suggested the potential application of anti-PGL-I and anti-LID-1 serology in monitoring the effectiveness of MDT (Cho et al. 2001, Duthie et al. 2011, Rada et al. 2012). Extending that data, in the current study, antibody responses typically declined during MDT. An exception was observed among patients who developed T1R or T2R during MDT, among which no decline in anti-LID-1 antibody levels was observed. In agreement, a study among 12 MB Filipino patients showed maintenance of high anti-LID-1 antibody titres in MB patients who developed T1R (Spencer et al. 2012). In the current study seroreactivity in paired samples of nonreactional MB patients before and after MDT confirmed the decline of anti-LID-1 antibodies levels. Similarly, in MB patients diagnosed for leprosy during T1R and T2R, a significant drop of anti-LID-1 antibody levels during MDT was observed. Therefore the maintenance of high antibody levels even during MDT suggests a prognostic role for anti-LID-1 serology in T2R leprosy reactions.

In an attempt to minimise potential confounding factors, the serologic reactivity of MB patients was stratified according to the type of leprosy reaction (T1R or T2R) and time of occurrence of reactions (at diagnosis or during MDT). These analyses allowed us to compare serologic reactivity without the influence of MDT among patients who developed T1R or T2R at diagnosis and in nonreactional patients. We also compared the seroreactivity among different groups of patients under the effect of MDT testing patients who developed T1R or T2R during treatment and nonreactional patients. Although we have monitored 50 well-characterised patients, the complexities of leprosy clinical outcomes at both the time of presentation and during treatment resulted in stratification of patients into small subgroups that somewhat limits interpretation. Regardless, our results indicate compelling differences associated with clinical outcome and certainly support validation with larger sample numbers. Large-scale valuations of new treatment strategies such as uniform trial MDT designed to investigate the use of a single treatment scheme with rifampicin, dapsone and clofazimine for six months for both PB and MB patients (Gonçalves et al. 2012) would appear best suited to provide the large number of well-characterised and monitored patients necessary to validate conclusions.

Anti-PGL-I antibody levels in MB patients who developed T1R/T2R at diagnosis were similar to the levels observed in reaction-free patients. Accordingly, a previous study of our research group did not detect differences in anti-PGL-I IgM and IgG antibodies in patients who developed T1R or T2R at diagnosis compared to patients who were reaction-free at diagnosis and to healthy endemic controls (Stefani et al. 1998). Furthermore, another study with Nepali patients and healthy controls from the United Kingdom also confirmed these results (Weir et al. 1998).

Results of serology of paired serum samples in MB patients collected in the presence and absence of T1R/T2R showed variable serological profiles with a declining trend in antibody levels after the reactional episode. Comparative analyses of seroreactivity in MB patients showed that high anti-LID-1 antibody levels indicated higher susceptibility to develop T2R at diagnosis or during MDT and these results merit further examination in expanded studies.
